# Development of a prediction model for facilitating the clinical application of transcranial color-coded duplex ultrasonography

**DOI:** 10.1186/s12880-024-01233-4

**Published:** 2024-03-05

**Authors:** Jieyu Duan, Pengfei Wang, Haoyu Wang, Wei Zhao

**Affiliations:** 1 Department of Ultrasound, The First Hospital of Hebei Medicine University, 89 Donggang Road, Yuhua District, 050030 Shijiazhuang City, Hebei Province China; 2 Department of neurosurgery, The Third Hospital of Hebei Medicine University, 050051 Shijiazhuang City, Hebei Province China

**Keywords:** Ultrasonography, Cerebrovascular diseases, Thickness, Temporal window, Age

## Abstract

**Background:**

Transcranial color-coded duplex ultrasonography (TCCD) is an important diagnostic tool in the investigation of cerebrovascular diseases. TCCD is often hampered by the temporal window that ultrasound cannot penetrate. Rapidly determine whether ultrasound can penetrate the temporal window in order to determine whether to use other acoustic windows to complete the examination process. In this study, Skull thickness can be measured simultaneously during TCCD examination, which makes it possible to use skull thickness to rapidly determine whether the temporal window is penetrated by ultrasound.

**Methods:**

This retrospective study included 301 patients with clinical symptoms of cerebrovascular diseases. These 301 patients were divided into an impenetrable temporal window (ITW) group and a penetrable temporal window group according to the results of the TCCD examination.

**Results:**

The area under the receiver operating characteristic (ROC) curve (AUC) for skull thickness was 0.887 (cutoff value 1.045 cm). Following multivariate logistic regression, sex, age, and skull thickness were used to develop a nomogram. The AUC for the nomogram was 0.923 (cutoff value 0.407).

**Conclusions:**

The skull thickness at the temporal window was measured by ultrasound, which was convenient and accurate. The probability of ITW in females was higher than that in males, and it increased with age. In this study, a prediction model incorporating sex, age and skull thickness could predict ITW probability well. If the patient’s temporal window was rapidly predicted as an ITW, other acoustic window examinations were used to complete the TCCD examination process to optimize the TCCD examination process of cerebrovascular diseases and facilitate the popularization of TCCD in clinical application.

## Background

At present, the disability rate and mortality rate of cerebrovascular diseases are relatively high [1–3]. Safe and effective diagnosis of intracranial vascular lesions in patients and targeted intervention measures are of great significance for delaying the disease course and the progression of the patient’s disease [4–6]. Transcranial color-coded duplex ultrasonography (TCCD), which can be very successful in identifying the intracranial arteries in older people, can intuitively show the intracranial vascular structure and blood flow status in real time, providing more comprehensive and accurate intracranial hemodynamic information [7, 8]. In some patients, satisfactory TCCD examination results cannot be obtained. The important factor is hampered by the temporal window that ultrasound cannot penetrate [9, 10]. In past examinations, sonographers decided whether to abandon the temporal window examination of cerebral arteries and to instead choose other acoustic window examinations based on their individual clinical experience. There is no standard for this operation. Existing studies had used computed tomography (CT) to study the relationship between skull thickness and ultrasound penetrativity [11, 12]. In our study, Skull thickness can be measured simultaneously during TCCD examination.

## Materials and methods

### Patient selection

This study was approved by our hospital ethics committee, and the requirement for informed consent was waived due to the retrospective nature of this study (grant number, 20,220,740). This retrospective study included 301 patients with clinical symptoms of cerebrovascular diseases in the neurology department and mental health department. The cases ranged from August 2021 to August 2022. The mean age was 67.5 ± 8.50 (50–92). According to the results of TCCD examination, these 301 patients were divided into 133 patients in the impenetrable temporal window (ITW) group and 168 patients in the penetrable temporal window (PTW) group. Impenetrable was defined as no signal being detected from any of the cerebral arteries [9, 11]. Penetrable was defined as successful display of any one of the cerebral arteries.

The inclusion criteria were as follows: (1) age ≥ 50 years and (2) complete TCCD results and clinical data. The exclusion criteria were as follows: (1) severe liver dysfunction or severe kidney dysfunction. (2) Patients who could not cooperate with the TCCD examination.

### TCCD examinations

**Fig. 1:**
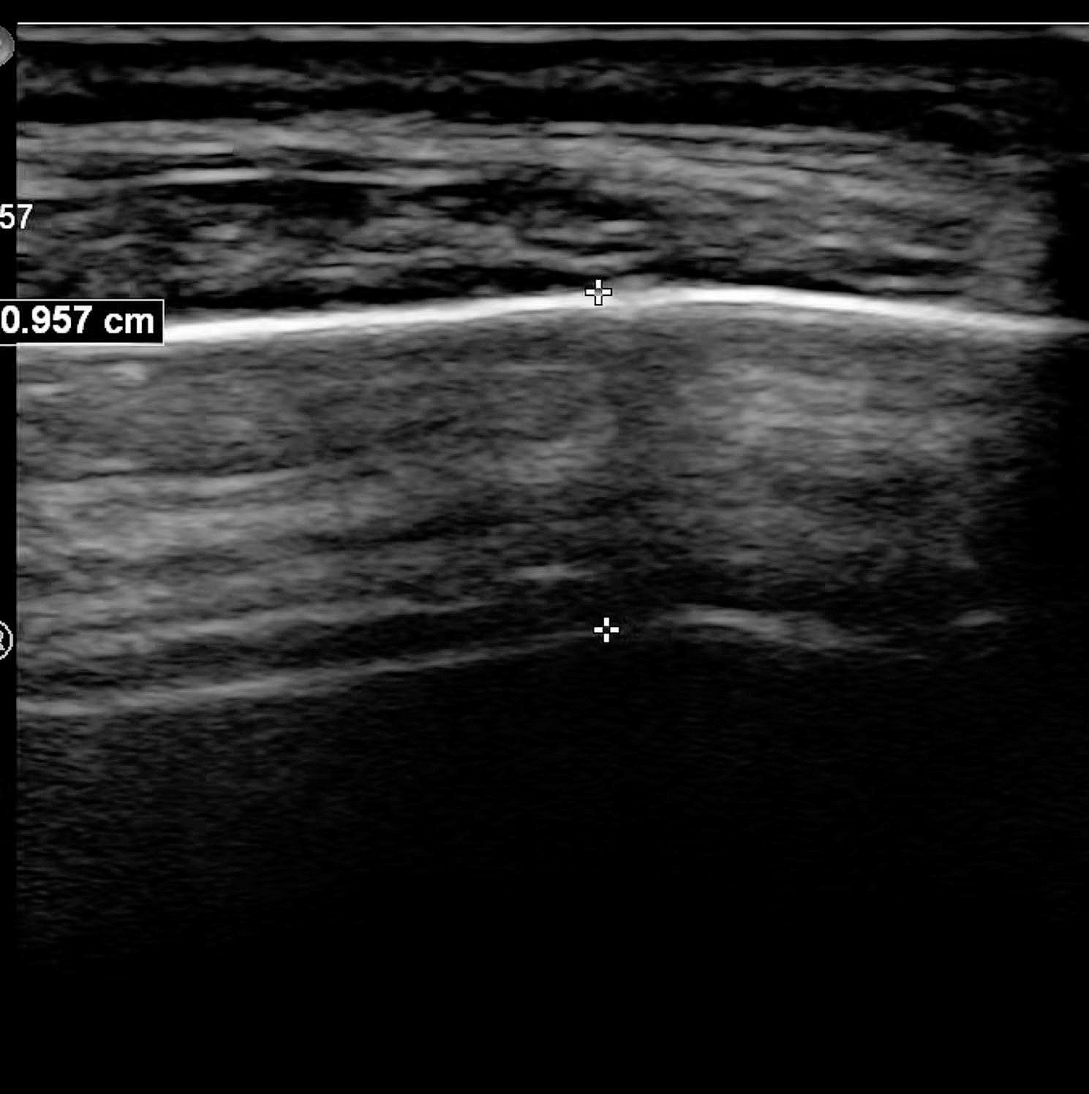
A Two-dimensional image of the skull thickness measured at the temporal window Skull thickness value = 0.957 cm

All patients were examined by using the Philips EPIQ7C (Philips Ultrasound, Inc. Bothell, Washington, USA, 2020) ultrasonic diagnostic instrument. The examination conditions were set to the transcranial Doppler mode (TCD) (image depth = 10 cm, color gain = 50%, dynamic range = 60). The X5-1 (xMATRIX array, 5 to 1 MHz) probe was placed at the temporal window to examine the cerebral arteries, such as the anterior cerebral artery, middle cerebral artery, and posterior cerebral artery, and to observe artery blood flow. The peak systolic velocity was recorded. The transtemporal window consists of an anterior, middle, and posterior window. However, in practice, there is usually only one useful window [10]. The temporal bone window is the thinnest area of the lateral skull located closest to the ear [11]. In our study, the temporal window extends from the cephalad to the zygomatic arch between the tragus and lateral wall of the orbit. This area at the “temple” can be palpated as a 3–4 cm diameter depressed area in the skull [13]. Skull thickness was the distance between the two layers of strong echoes from the periosteum to the dura, measured in centimeters at the thinnest part of the skull at the temporal window (Fig. 1). The L12-3 (broadband linear array, 12 to 3 MHz) probe was used to measure skull thickness by using superficial organ examination conditions (image depth = 4 cm, gain = 44%, dynamic range = 57). The thickness of the left and right sides was measured multiple times separately, using data from the relatively thinnest value. For patients with ITW, other acoustic windows were used to complete the TCCD examination process. All examinations were performed by the same ultrasound physician with over twenty years of work experience. The ultrasound physician was blinded to the clinical and demographic data of the study patients during the data collection and analysis phase.

### Statistical analysis

Continuous variables conforming to a normal distribution are presented as the mean ± SD, and the comparison between groups was performed by two independent samples *t* tests. Nonnormally distributed data are expressed as median and interquartile-range values, and comparisons between groups were performed using the nonparametric rank sum test. Categorical variables were expressed as frequencies and proportions (%), and comparisons between groups were performed using the χ ^2^ test. The pROC package of R software was used for receiver operating characteristic (ROC) curve analysis. Multivariate logistic regression analysis was used. The “RMS” package was used to develop the nomogram. Calibration curve analysis and decision curve analysis (DCA) were used to evaluate the accuracy, consistency and clinical utility of the predictive model. For all statistical tests, *P* < 0.05 was indicative of statistical significance. All statistical analyses were carried out using R software (version 4.0.2).

## Results

The patients’ baseline characteristics, including sex, age and skull thickness, are given in Table 1.

**Table 1:** Baseline characteristics

Variables	Total (*n* = 301)	Impenetrable temporal window (*n* = 168)	Penetrable temporal window (*n* = 133)	*P*
Sex, *n* (%)				< 0.001
Female	181 (60)	103 (77)	78 (46)	
Male	120 (40)	30 (23)	90 (54)	
Age, Mean ± SD(year)	67.5 ± 8.50	68.8 ± 8.68	66.46 ± 8.23	0.018
Thickness, Median (Q1,Q3)(cm)	1.02 (0.94, 1.08)	1.08 (1.06, 1.12)	0.97 (0.90, 1.02)	< 0.001

Note: Univariate analysis showed that sex, age and skull thickness were statistically correlated with the ultrasound penetrativity (*P* < 0.05). Impenetrable was defined as no signal being detected from any of the cerebral arteries. Penetrable was defined as successful display of any one of the cerebral arteries. Thickness was measured in centimeters at the thinnest part of the skull at the temporal window

**Fig. 2:**
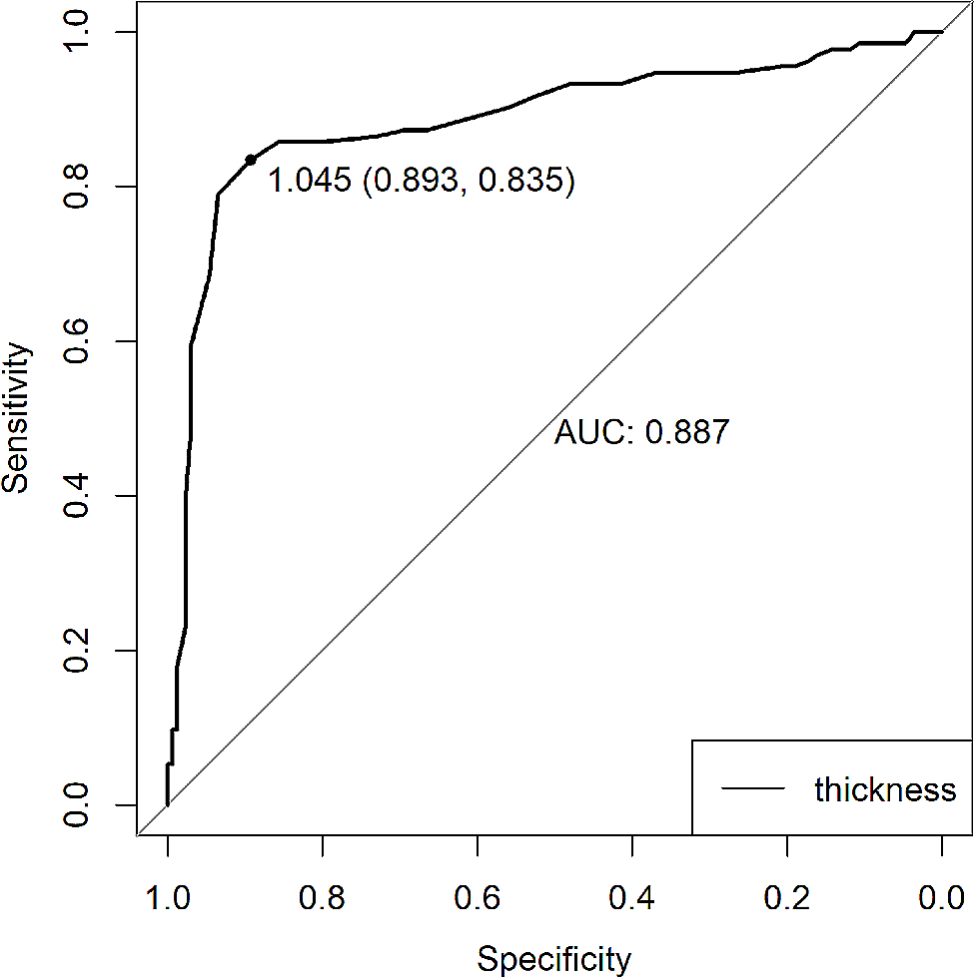
Receiver operating characteristic curve analysis for the thickness

. The univariate analysis showed that sex ( *P* < 0.001), age ( *P* = 0.018) and skull thickness ( *P* < 0.001) were significantly different between the ITW group and PTW group. The area under the ROC curve (AUC) for skull thickness was 0.887 (cutoff value 1.045, specificity 0.893, sensitivity 0.835, 95% confidence interval (CI) of the AUC 0.8455–0.9294) (Fig. 2).

The patients were divided into a skull thickness ≥ 1.045 cm group and a skull thickness < 1.045 cm group. The results of the χ ^2^ test showed *P* < 0.001 (Table 2).

**Table 2:** Comparison of ultrasound penetrativity grouped by thickness

Group	Impenetrable temporal window (*n* = 133)	Penetrable temporal window (*n* = 168)	χ ^2^	*P*
Thickness ≥ 1.045 cm	111(86.0%)	18(14.0%)	160.401	< 0.001
Thickness < 1.045 cm	22(12.8%)	150(87.2%)		

Note: The area under the ROC curve (AUC) for skull thickness was 0.887 (cutoff value 1.045 cm). Impenetrable was defined as no signal being detected from any of the cerebral arteries. Penetrable was defined as successful display of any one of the cerebral arteries

**Fig. 3:**
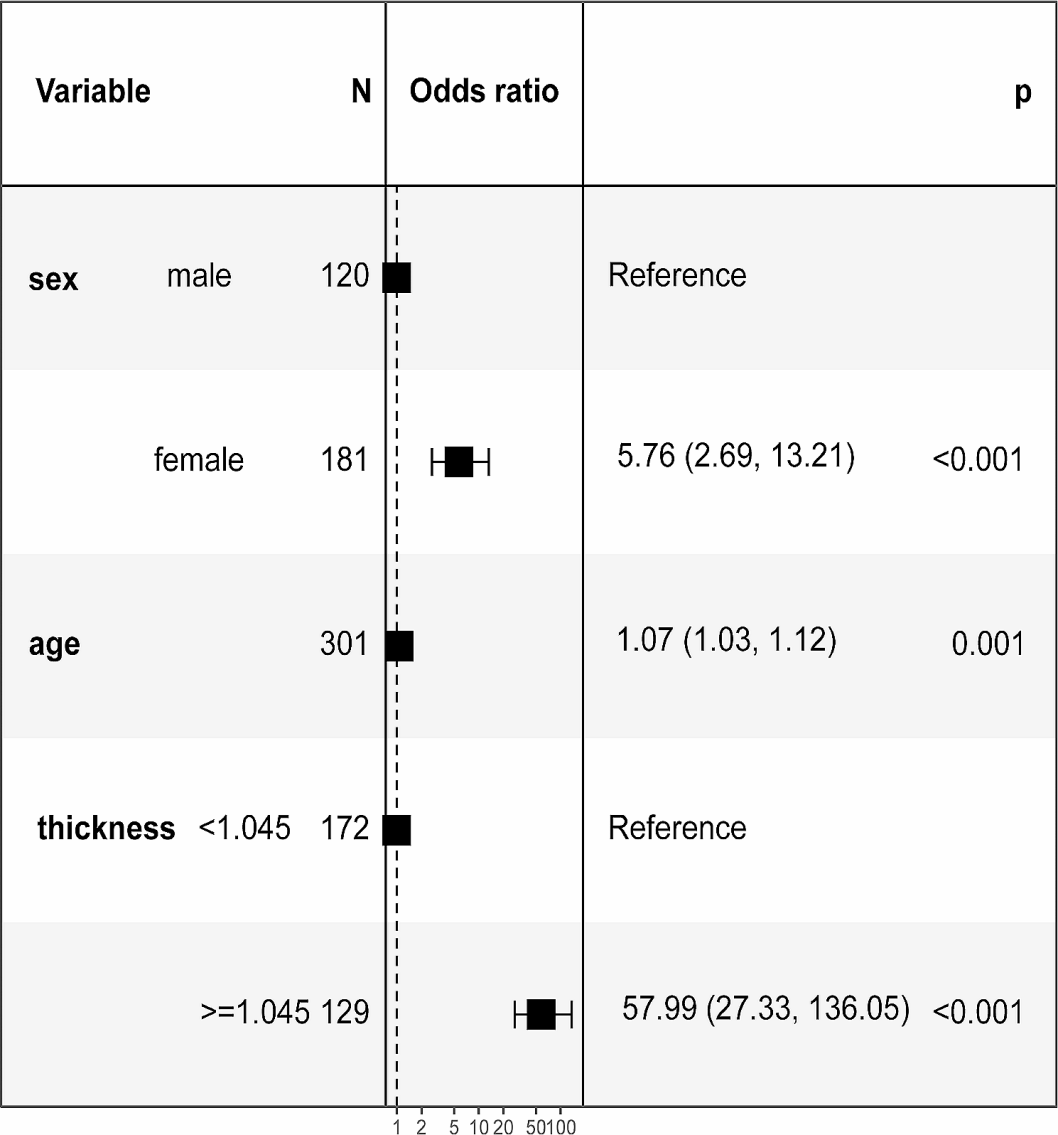
Multivariate regression analysis for the predictive factors of an impenetrable temporal window

. The multivariate logistic regression for sex, age, and skull thickness showed that sex (OR = 5.76, 95% CI, 2.69–13.21, *P* < 0.001), age (OR = 1.07, 95% CI, 1.03–1.12, *P* = 0.001), and skull thickness (OR = 57.99, 95% CI, 27.33-136.05, *P* < 0.001) were independent predictors for ITW (*P* < 0.05) (Fig. 3).

**Fig. 4:**
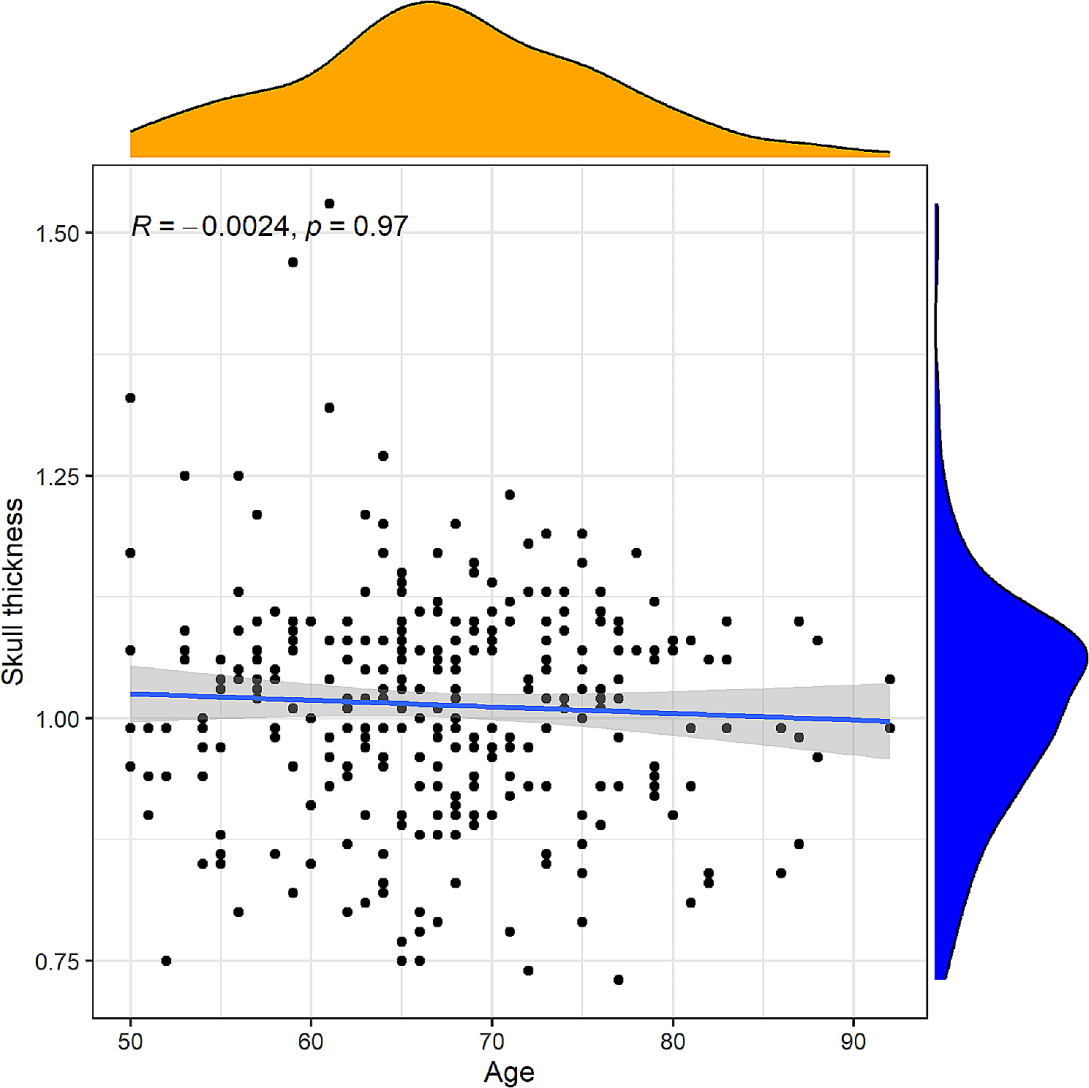
Correlation between age and skull thickness

**Fig. 5:**
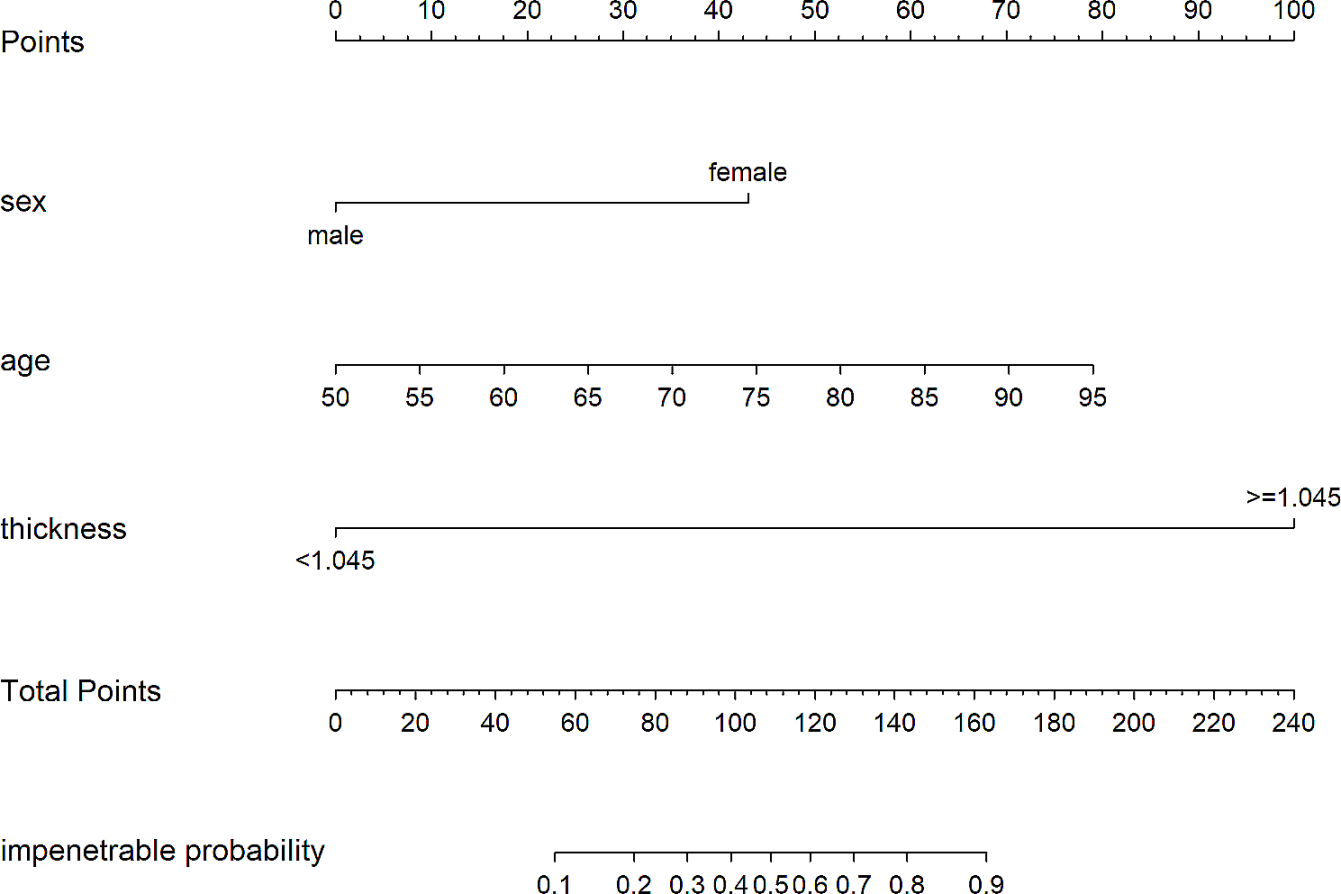
A nomogram predicting impenetrable temporal window probability Impenetrable probability is defined as the impenetrable temporal window probability

The linear regression correlation between age and skull thickness (*r* = -0.0024; *P* = 0.97) is shown in Fig. 4. Based on the multivariate analysis results, the prediction model incorporating sex, age and skull thickness was developed as a nomogram (Fig. 5). The specific scoring table of the nomogram is shown in Table 3.

**Table 3:** Values assigned to each variable in the nomogram

Variable	Assignment	Score
Sex	female	43
	male	0
Age	50	0
	55	9
	60	18
	65	26
	70	35
	75	44
	80	53
	85	61
	90	70
	95	79
Thickness	< 1.045	0
	>=1.045	100
Total score	55	ITW probability = 0.1
	75	ITW probability = 0.2
	88	ITW probability = 0.3
	99	ITW probability = 0.4
	109	ITW probability = 0.5
	119	ITW probability = 0.6
	130	ITW probability = 0.7
	143	ITW probability = 0.8
	163	ITW probability = 0.9

Note: Impenetrable temporal window (ITW)

**Fig. 6:**
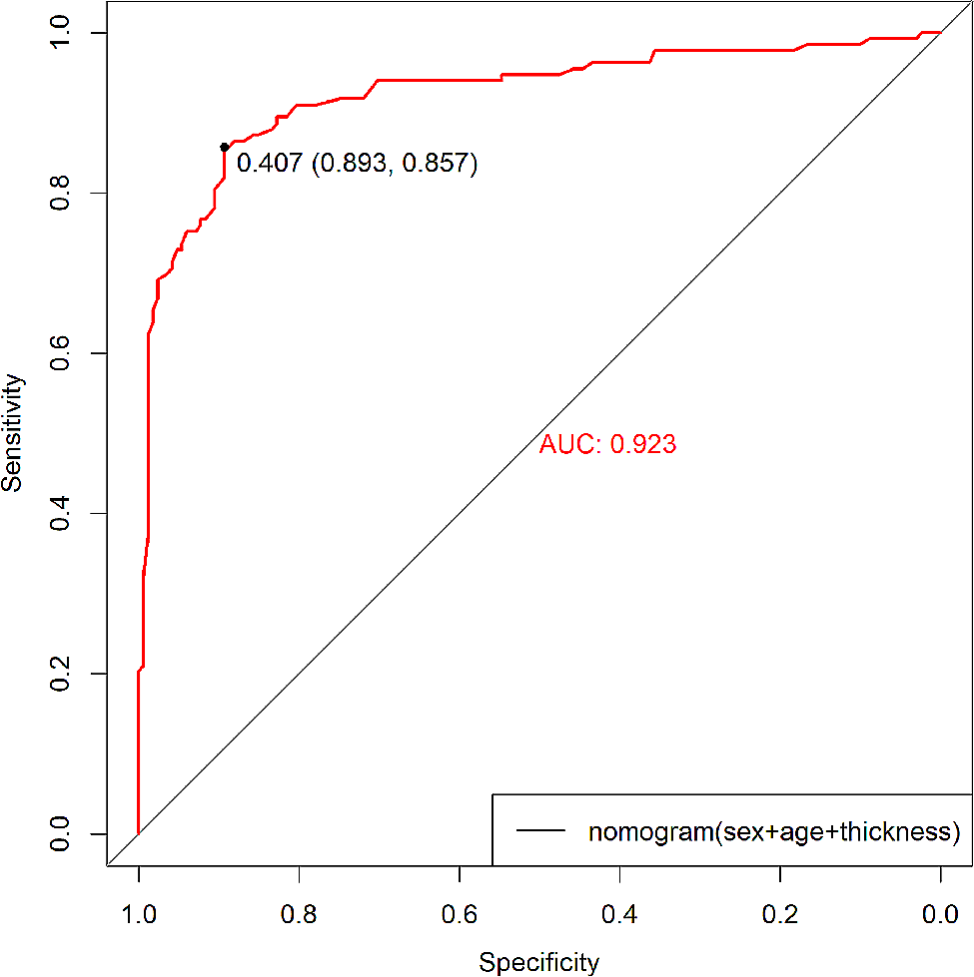
Receiver operating characteristic curve analysis for the nomogram AUC of the nomogram ROC was 0.923

**Fig. 7:**
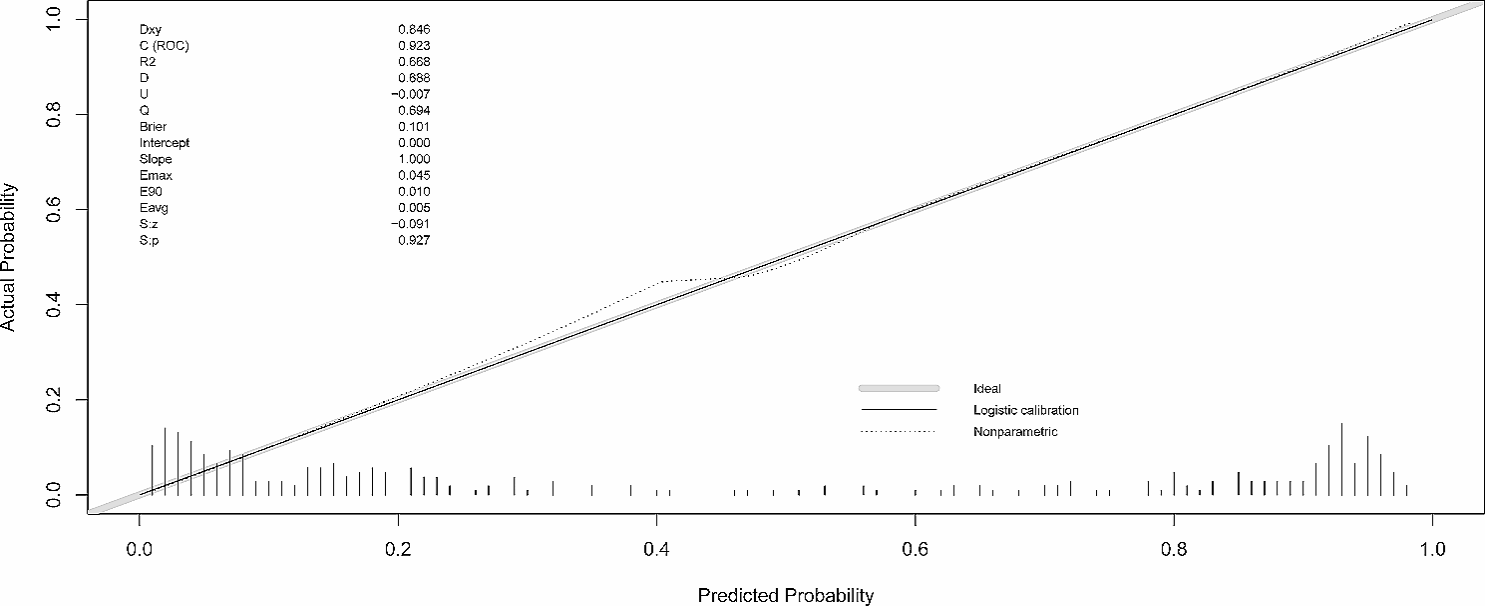
Calibration curve of the nomogram

**Fig. 8:**
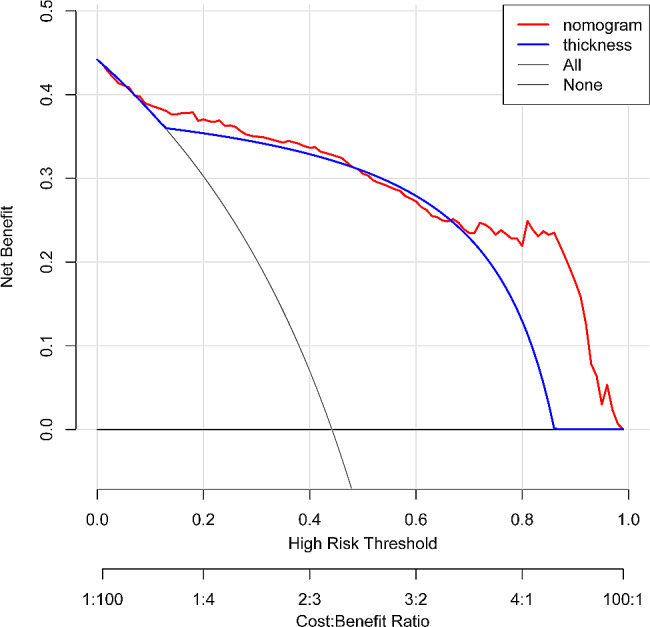
Decision curve analysis for the nomogram and the thickness Net benefit range of the thickness was approximately [0.13–0.86]. Net benefit range of the nomogram was approximately [0.02–0.98]

. The AUC for the nomogram was 0.923 (cutoff value 0.407, specificity 0.893, sensitivity 0.857, 95% CI, 0.8901–0.9564) **(**Fig. 6). The calibration curve for predicting ITW probability is shown in Fig. 7. The Hosmer‒Lemeshow test yielded a nonsignificant statistic (*P* = 0.927). The DCA curve net benefit range of skull thickness for predicting ITW probability was approximately [0.13–0.86] (Fig. 8). The DCA net benefit range of the nomogram was approximately [0.02–0.98] (Fig. 8).

## Discussion

Both TCD and TCCD are important methods to detect intracranial artery lesions. TCD is an imaging technique based on Doppler spectrum for analyzing arterial functional status. No two-dimensional images are obtained, and the examination requires a skilled examiner. Because the angle between the acoustic beam and blood flow direction cannot be accurately corrected, there are many variations in normal intracranial blood vessels; this further increases the difficulty of examination [14, 15]. TCCD can display the intracranial blood flow and hemodynamic status by color Doppler imaging. TCCD can also directly observe the lesion location, size and abnormal blood flow state of the vascular mass [16, 17]. With the increase in the number of cerebrovascular disease patients, TCCD examination has been widely used in the clinical diagnosis of various diseases [18, 19].

TCD can be a time-consuming investigation in case of an inadequate sign in patients with insufficient temporal bone window [11]. In a survey reviewing conventional TCD results from 60 laboratories in the United States, the percentage of failure to access the temporal window ranged between 0% and 65% (mean, 16%) [20]. Visualization of all major intracranial arteries was possible in only one-third of female patients older than 60 years of age [9] The reason why ultrasound cannot penetrate the temporal window in some patients is that the ultrasonic signal is severely attenuated, which affects the quality of the ultrasonic image and the detection result [21]. A study evaluated the association between transtemporal window failure on TCCD and radiologic findings on CT of the skull. On logistic regression, age, female sex, and temporal bone thickness (OR = 3.04, *P* < 0.001) were independent predictors of transtemporal window failure [22].

During the TCCD examination, it is very important to determine that the temporal window is impenetrable and not that the intracranial arteries cannot be displayed due to unskilled operation.

If the patient’s temporal window is predicted as ITW, other acoustic window examinations will be used to complete the TCCD examination process. To judge whether the ITW rate is efficient or not directly affects the TCCD examination process. In this study, the L12-3 probe was used to measure the skull thickness of the temporal window, and this technique was convenient with its high measurement accuracy and real-time display. The results of this study can improve the efficiency of TCCD.

The univariate analysis in this study showed that skull thickness, sex, and age were statistically correlated with ITW (Table 1). Sex was significantly different between the ITW group and PTW group (77% vs. 23%, *P* < 0.001). The proportion of females was higher in the ITW group than in the PTW group. Age was significantly different between the ITW group and PTW group (68.8 vs. 66.46, *P* = 0.018). The Age in the ITW group was higher than that in the PTW group.

The AUC for skull thickness was 0.887 (95% CI: 0.8445–0.9294) (Fig. 2), indicating high accuracy. The cutoff was 1.045 cm. The patients were divided into two groups according to the cutoff value, and there was a significant difference between the skull thickness ≥ 1.045 cm group and the skull thickness < 1.045 cm group (86.0% vs. 12.8%, *P* < 0.001) (Table 2). The proportion of ITW was higher in the skull thickness ≥ 1.045 cm group than in the skull thickness < 1.045 cm group.

The multivariate logistic regression analysis identified sex, age, and thickness as independent predictors (*P* < 0.05) (Fig. 3). The OR value of skull thickness was 57.99, which was the largest of the three independent predictors. Skull thickness was a reliable independent predictor for ITW. The OR value of sex was 5.76, indicating that females had a worse effect of ITW than males. The OR value of age was 1.07, indicating that the probability of ITW increased with age. In this study, the probability of ITW in females was higher than that in males, and it increased with age.

The most likely explanation for temporal window failure is hyperostosis of the temporal bone, which is influenced by age, sex, and thickness [22–24]. With an increase in the aging population worldwide, the prevalence of osteoporosis increases at an alarming rate in both males and females, irrespective of their ethnicity, and elderly women are prone to osteoporosis [25, 26]. Because severe liver dysfunction and severe kidney dysfunction are associated with osteoporosis [27, 28], patient selection in this study was excluded.

No linear correlation between age and skull thickness is shown in Fig. 4. Furthermore, based on the multivariate analysis results, a prediction model incorporating the independent predictors, sex, age and skull thickness, was developed as the nomogram (Fig. 5). The specific scoring table of the nomogram is clearly shown in Table 3 for convenience in clinical application. This study showed that the nomogram improved predictive accuracy (the AUC for the nomogram increased to 0.923. 95% CI, 0.8901–0.9564) (Fig. 6). The calibration curve of the nomogram demonstrated good agreement between the probability as predicted and the actual probability. The Hosmer‒Lemeshow test yielded a nonsignificant statistic (*P* = 0.927), which suggested that there was no departure from perfect fit (Fig. 7). The calibration curve showed that the nomogram had an optimal ability to predict ITW probability. The DCA curve net benefit range of skull thickness for predicting ITW probability was approximately [0.13–0.86] (Fig. 8). The DCA curve net benefit range of the nomogram was approximately [0.02–0.98] (Fig. 8). Decision curve analysis demonstrated that the nomogram had a higher clinical application value than skull thickness.

In our study, Skull thickness was measured simultaneously during TCCD examination, which makes it possible to use skull thickness to rapidly determine whether the temporal window was penetrated by ultrasound. The skull thickness values of all patients included were different from those measured by ultrasound in this study ((1.02(0.94, 1.08)) and CT in other studies ((3.1 ± 0.9 mm) [11]; (0.27 ± 0.06) [12]). However, the temporal soft tissue thickness value (0.56 ± 0.12) measured by ultrasound in He et al. [12] study was more than twice the temporal bone thickness value (0.27 ± 0.06) measured by CT. It can be seen that, combined with the content shown in Fig. 1, the measurement results of skull thickness will be different due to the different principles of ultrasound and CT imaging.

Our study had the following limitations: The measurement position of the temporal window skull thickness needs to be more accurate and relatively fixed. Our findings are based on retrospective data from a single centre, and there is a possibility of unmeasured covariates.

## Conclusion

In this study, the skull thickness of the temporal window was measured by ultrasound, which was convenient and accurate. Skull thickness was an independent predictor for ITW. The probability of ITW in females is higher than that in males, and it increases with age. A prediction model incorporating sex, age and skull thickness could predict ITW probability well. If the patient’s temporal window was rapidly predicted as ITW, other acoustic window examinations were used to complete the TCCD examination process to optimize the TCCD examination process of cerebrovascular diseases and facilitate the clinical application of TCCD.

## Data Availability

The datasets used and/or analysed during the current study are available from the corresponding author on reasonable request.

## Abbreviations

TCCD: Transcranial color-coded duplex ultrasonography
CT: Computed tomography
TCD: Transcranial Doppler ultrasound
ITW: Impenetrable temporal window
PTW: Penetrable temporal window
ROC: Receiver operating characteristic
AUC: Area under the curve
DCA: Decision curve analysis
